# Impact of mammalian cell culture conditions on monoclonal antibody charge heterogeneity: an accessory monitoring tool for process development

**DOI:** 10.1007/s10295-019-02202-5

**Published:** 2019-06-07

**Authors:** Bernhard Sissolak, Nico Lingg, Wolfgang Sommeregger, Gerald Striedner, Karola Vorauer-Uhl

**Affiliations:** 1 Department of Biotechnology, University of Natural Resources and Life Sciences (BOKU), Vienna, Austria; 2 Austrian Centre of Industrial Biotechnology GmbH (ACIB), Vienna, Austria; 3 Bilfinger Industrietechnik Salzburg GmbH, Salzburg, Austria

**Keywords:** Product quality, Recombinant mAbs, Charge heterogeneity determination, Mammalian cell culture, CHO

## Abstract

Recombinant monoclonal antibodies are predominantly produced in mammalian cell culture bioprocesses. Post-translational modifications affect the micro-heterogeneity of the product and thereby influence important quality attributes, such as stability, solubility, pharmacodynamics and pharmacokinetics. The analysis of the surface charge distribution of monoclonal antibodies provides aggregated information about these modifications. In this work, we established a direct injection pH gradient cation exchange chromatography method, which determines charge heterogeneity from cell culture supernatant without any purification steps. This tool was further applied to monitor processes that were performed under certain process conditions. Concretely, we were able to provide insights into charge variant formation during a fed-batch process of a Chinese hamster ovary cell culture, in turn producing a monoclonal antibody under varying temperatures and glucose feed strategies. Glucose concentration impacted the total emergence of acidic variants, whereas the variation of basic species was mainly dependent on process temperature. The formation rates of acidic species were described with a second-order reaction, where a temperature increase favored the conversion. This platform method will aid as a sophisticated optimization tool for mammalian cell culture processes. It provides a quality fingerprint for the produced mAb, which can be tested, compared to the desired target and confirmed early in the process chain.

## Introduction

Recombinantly produced monoclonal antibodies (mAbs), as well as biosimilars, are key products in today’s pharmaceutical industry [1, 2]. Post-translational product modifications induced by chemical and enzymatical intra- and extracellular mechanisms during the production process lead to micro-heterogeneity of mAbs, in turn affecting product characteristics (e.g., efficacy, safety, pharmacodynamics and pharmacokinetics) [3]. The recombinant cell line, the culture media and the process settings affect these quality attributes [4, 5]. During process development, it is important to ensure a reproducible, distinct and preferably homogenous pattern of the product. For the establishment of biosimilars, it is important to match the characteristics of the originator product [6]. The effects of various extra- and intracellular influences on different aspects of product quality have been evaluated in great detail. For instance, *N*-glycosylation is by far the best-studied quality attribute and there are several strategies available for glycosylation control [7].

One additional important measure of mAb heterogeneity is the distribution of surface charge variants. Due to numerous modifications, the net surface charge of mAbs can be altered [8–11]. Charge species with a lower isoelectric point (pI) than the main fraction of the product are defined as acidic variants and generated by sialylation, deamidation of asparagine and glutamine, glycation and other mechanisms. Glycation, for instance, is a non-enzymatic reaction where a reducing sugar molecule, most commonly glucose, is covalently bound to a reactive amino group [12]. Basic variants are defined as species with a higher pI than the main fraction and generated by incomplete C-terminal lysine clipping of the heavy chains, as well as by fragmentation and aggregation [13]. Several studies indicate that mAb variants can lead to varying biological responses [14–16]. For instance, it was shown that the basic variants exhibited an increased binding to the FC and the neonatal receptor, indicating an increased half-life [15]. Another study reported that only a few specific variants of the tested mAb had a statistical relevant impact on the cell proliferation assay [10]. Hence, to know and understand the mechanism behind the charge heterogeneity is of particular importance.

Common analytical methods for the determination of charge heterogeneities of mAbs are capillary isoelectric focusing (cIEF) and ion exchange chromatography (IEX) [17]. Both methods are widely used in various applications [18], but IEX methods, using a salt gradient elution, are recognized as the gold standard and routinely in use [19–22]. The major limitation of IEX is when using a salt buffer system to coerce the user to adapt it for every new kind of mAb. However, the use of pH gradients was shown to be product-independent [23] and recently, a cation exchange chromatography (CEX) method with a linear pH gradient for the determination of charge heterogeneity of mAbs was published [24]. This technique was shown to be robust, exhibit a high resolution [25], result in similar precisions compared to imaged cIEF [26] and be scalable for semi-preparative purposes [14, 15].

Monitoring and controlling of product quality are required for the whole production chain [27]. The successful application of process analytical technology (PAT) and quality by control (QbC) to bioprocesses [28] requires reliable and unbiased product quality data over the time course of a fermentation process. Samples taken from crude culture supernatants should be analyzable with a minimum of manipulation. In this respect, pre-purification of relevant samples would possibly falsify the results [29–31]. Moreover, the avoidance of purification steps reduces the workload, while the method becomes more applicable as a process-monitoring tool and allows for decision making early in the process chain.

In this work, we aimed to adjust the method developed by Lingg et al. [24] for the measurement of charge heterogeneity directly from cell culture supernatants without prior purification or additional sample manipulation. This approach offers the possibility to assess any quality changes already in the early stages of cell line, media and process development. Eventually, the derived data enable advanced process characterization and monitoring. In the first part of this manuscript, we explain the applicability of CEX separation for the analysis of crude culture supernatants and evaluate the influence of matrix effects. In the following, it is used as a process-monitoring tool for a model antibody, expressed in Chinese hamster ovary (CHO) cells, within an experimental setting, while varying glucose concentration in the feed media and cultivation temperature. Process relevant samples were analyzed by CEX to study the impact of these variations on mAb charge heterogeneity.

## Materials and methods

### Fed-batch experiments

A recombinant CHO monoclonal cell line, generated by the *Rosa26* bacterial artificial chromosome expression strategy [32], producing an antitumor necrosis factor (TNF) alpha IgG1, was used (Antibody Lab GmbH, Austria). The cell line originated from the host cell CHO-K1 (ATCC CCL-61), which was serum-free adapted for prior use. A working cell bank of the recombinant cell line with 5 × 10^6^ cells per vial was used as the starting material for all experiments. The cells were thawed in chemically defined culture medium (Dynamis AGT, A26175, Thermo Fisher Scientific, USA) supplemented with 8 mM l-glutamine (25030081, Sigma Aldrich, Germany), 3 mL/L phenol red solution (RNBD642, Sigma Aldrich, Germany), 1:1000 anti-clumping agent (0010057DG, Thermo Fisher Scientific, USA) and 1 mg/mL G418 (10131027, Thermo Fisher Scientific, USA).

The culture was subsequently passaged three times (every 3–4 days) in the above-mentioned media without G418 and anti-clumping agent and used as the starting material for the inoculation of the batch with a starting cell density of 2.5 × 10^5^ cells/mL. The fed-batch cultivations were performed in shake flask (#431147, Corning, USA) with a starting volume of 300 mL. As batch medium, the culture medium was additionally supplemented with 0.1% (v/v) Antifoam C (A8011, Sigma Aldrich, Germany) to represent typical large-scale cultivation conditions. Within the experimental setup, the parameters of temperature and glucose addition during the feed phase were changed. In this study, the feed (CHO CD EfficientFeed™ A AGT™ Kit, A1442002, Thermo Fischer Scientific, USA) was supplemented with 0.1% antifoam as well as additional 10, 20 or 30 g/L glucose, which will be referred to as Feed 1, Feed 2 and Feed 3, respectively. The pulse feeding started at day 3 and lasted until day 13. A linear feed rate was carried out with a total added feed volume of 33 vol% (v/v) with respect to the end volume. The process temperatures were changed at day 4–31 °C or 34 °C or remained constant at 37 °C.

An 11 mL sample was drawn each day for several offline analyses. The cultivations were terminated when the viability dropped below a threshold of 70%. All cultivations were conducted in a humidified CO_2_ incubator (Heracell™ VIOs 160i, Thermo Scientific, USA) at 5% (v/v) CO_2_ in ambient air, at the temperature defined in the experimental design with an orbital shaker (MaxQ 2000 CO_2_ Plus, Thermo Scientific, USA) at 200 rpm.

For the mock control fed-batch bioprocess, the host cell line was cultivated at a constant 37 °C with Feed 3 (+ 30 g/L glucose). The fed-batch was performed applying the same settings as mentioned above.

### All experiments were carried out in duplicates

For the LC–MS and the boronate affinity chromatography analysis, samples from a stirred tank reactor (*V* = 15 L) were used. The same procedure, cell line and parameters as stated above were utilized.

### Analytics

The total cell concentration (TCC) was determined by counting the cell nuclei using the particle counter Z2 (Beckman Coulter, USA). Therefore, an aliquot of the cell suspension was centrifuged for 10 min at 200*g* at room temperature. The cell pellet was resuspended in a 0.1 M citric acid monohydrate (C1909, Merck, Germany) and 2% (v/v) Trition X-100 (Merck, Germany) buffer. A minimum of 1 h later, an aliquot of the lysate was diluted with 9 mL of a 0.9% NaCl solution and measured.

Viability was measured by the trypan blue (K490, Amresco, USA) exclusion assay [33]. The viable cell concentration (VCC) was calculated by applying the viability to the TCC.

The product titer was determined by bio-layer interferometry (BLI) using Protein A tips (Octet System, QK, ForteBio, USA) as already described by [34].

The carbohydrates were measured via ion exclusion chromatography (HPX 87H, 300 × 7.8 mm, #1250140, BioRad, USA) on an Agilent 1200 series device (Agilent, USA). The column was tempered at 25 °C. The mobile phase was 5 mM sulfuric acid and the flow rate was 0.45 mL/min. The used detector was a refractive index detector tempered at 35 °C. The calibration range for d(+)-glucose was between 100 and 2000 mg/L. The chromatograms were evaluated with ChemStation software (Revision B.04.01, Agilent, USA).

Gel electrophoresis was performed with an Invitrogen NuPage™ 4–12% Bis–tris gel (NP0321BOX) in a Novex Mini-cell chamber (both Thermo Fisher Scientific, USA). A SeeBlue^®^ Plus2 pre-stained protein standard (LC5925, Thermo Fisher Scientific, USA) was used for size comparison. The samples were applied with 4 × sample buffer (NuPAGE LDS, NP0007, Thermo Fisher Scientific, USA), while the used running buffer contained 0.3% (w/v) Tris, 1.5% (w/v) glycine and 0.1% (w/v) SDS. Gels were run at 150–200 V. Adalimumab (Humira™, AbbVie, USA) was used as a reference.

Protein A purification was done via a Proteus Protein A mini spin column (PUR 006, Bio-Rad, USA) according to the manual instructions.

Glycated mAb species were determined via boronate affinity chromatography (BAC) (0013066, Tosoh Bioscience, Japan). Solvent A consisted of 50 mM EPPS (E9502, Merck, Germany), 10 mM Tris (65837, Fluka, USA) and 200 mM NaCl (S7653, Merck, Germany), which were adjusted to a pH 8.7 with 10 M NaOH. Solvent B was 500 mM sorbitol (85529, Merck, Germany) in Mobile Phase A. Chromatography was performed according to a previously published study [35].

For the peptide analysis, the samples were digested in gel and analyzed via LC–MS as previously published [36–38].

### Charge heterogeneity determination

For the method, as previously described in two publications [24, 25], a weak cation exchange resin (Dionex ProPac WCX-10 4 × 250 mm, 088768, Thermo Fisher Scientific, USA) was utilized. Due to the fact that supernatants were directly applied, a guard column (4 × 50 mm) was also installed (054994, Thermo Fisher Scientific, USA). Two complex, four-component buffers were used to ensure a highly linear pH gradient. The compounds were 3-morpholino-2-hydroxypropanesulfonic acid (MOPSO, M8389), 4-(2-hydroxyethyl)piperazine-1-ethanesulfonic acid (HEPES, H3375)), *N*,*N*-bis(2-hydroxyethyl)glycine (Bicine, B3879,), 3(cyclohexylamino)-2-hydroxy-1-propanesulfonic acid (CAPSO, C2278) and 3-(cyclohexylamino)-1-propanesulfonic acid (CAPS, C2632, all Merck, Germany). To ensure elution was only based on the pH shift, sodium chloride (S7653, Merck, Germany) was added to Buffer A according to Table 1 to obtain constant conductivity. The pH was adjusted with sodium hydroxide (Merck, Germany). The two different buffer systems used are listed in Table 1.

**Table 1:** Running (A) and elution (B) buffer compositions used in the CEX method

System	Buffer	HEPES	MOPSO	Bicine	CAPSO	CAPS	NaCl
pH 7–10.5	A (mM)	0.0	7.1	5.3	14.9	0.7	12.6
	B (mM)	0.0	14.6	4.9	1.4	7.1	0.0
pH 8–10.5	A (mM)	5.5	0.0	4.2	9.5	0.8	6.3
	B (mM)	0.0	0.0	10.5	2.5	7.0	0.0

In this study, supernatants were applied using a flow rate of 1.0 mL/min, while the injection volume was 100 µL. The elution gradient for both buffer systems was set to 0.07 pH/min. The chromatograms were evaluated with ChemStation software. As a reference, the same adalimumab standard as stated above was used. Statistical analysis was performed with SigmaPlot 13.0 software.

## Results and discussion

### Assessment of the method’s appropriateness concerning the cell culture matrix

Since the IEX method has already been thoroughly verified, this study is entirely focused on the adaptation for cell culture samples. Critical considerations were taken in terms of possible matrix effects and the qualitative evaluation of the resulting peak areas.

**Fig. 1:**
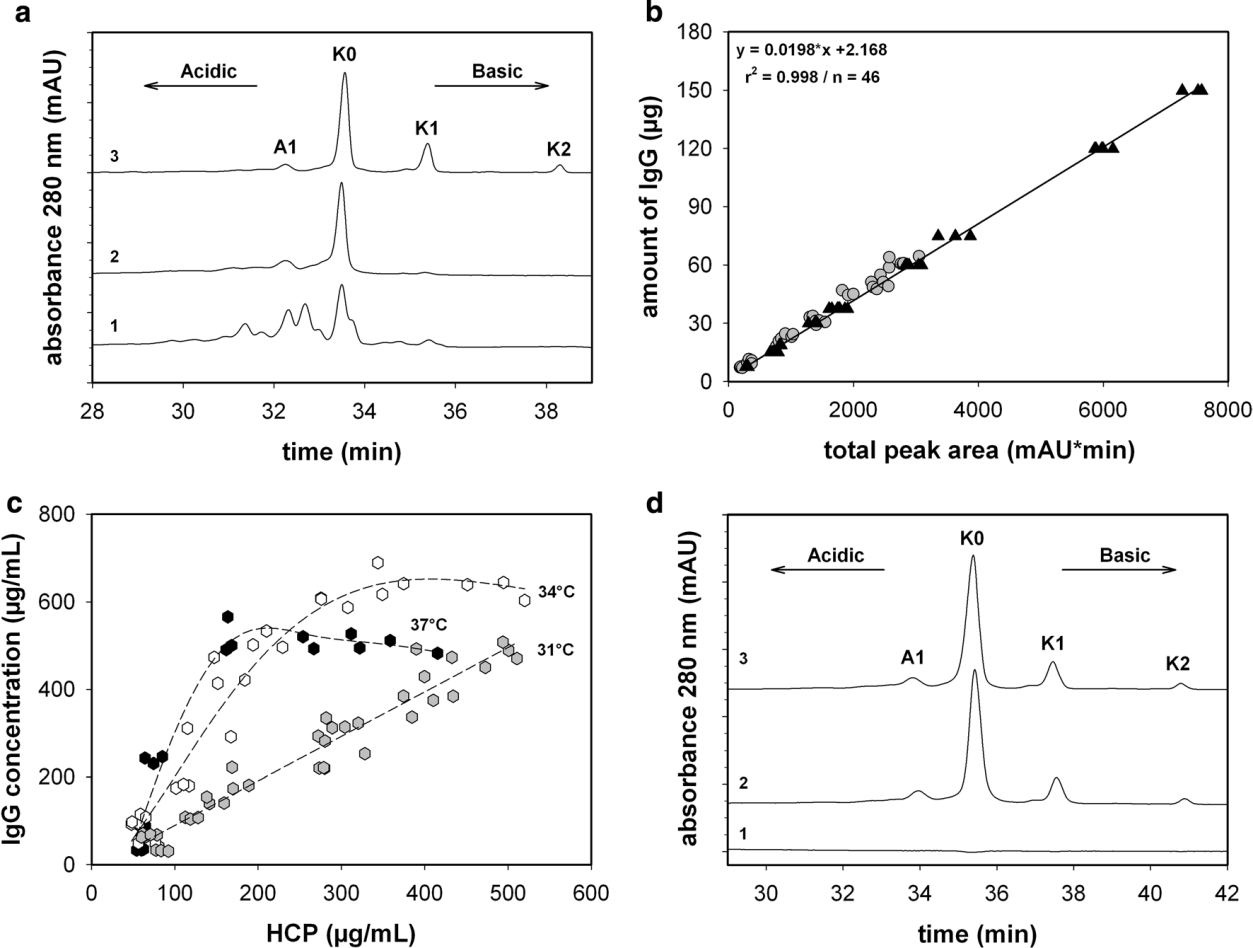
**a** HPLC-chromatogram of a recombinantly produced protein measured directly from the supernatant at harvest (1), the same sample but pre-purified via Protein A (2) and the adalimumab reference (3), using the pH 7 buffer system. **b** Amount of IgG as a function of the integrated total peak area for the shaker data [grey-filled circle] and control standards [filled triangle]. **c** Amount of host cell proteins (HCPs) and IgG of fed-batch experiments at 37 °C [filled hexagon], 34 °C [unfilled hexagon] and 31 °C [grey hexagon]. **d** CEX chromatogram of a mocking supernatant (1), a standard spiked into the same supernatant (2) and the standard (3). *K0* main variant, *K1* 1-lysine variant, *K2* 2-lysine variant, *A1* acidic variant without further characterization. Linear regression was performed on the adalimumab dataset

The recombinantly produced anti-TNF-alpha antibody was compared to a pharmaceutical adalimumab reference, which is a well-described mAb [21]. The reference standard analyzed by a CEX with a linear pH gradient exhibited a distinct peak distribution (see Fig. 1a). Due to the use of a strictly pH-dependent system, the charge variants were only separated according to their net surface charge, where acidic forms were eluted prior to the neutral and basic variants. Both buffer systems (pH 7–10.5 and pH 8–10.5) and different flow rates were tested in terms of their applicability for supernatants. The pH gradient itself was set to a constant slope of 0.07 pH/min. In conclusion, the pH 7 variant resulted in a better separation of the acidic species. It is obvious that the close proximity of the calculated pI of the mAb (8.60) and the starting pH conditions affect the separation profile of the acidic isoforms. The tested flow rates, between 0.5 and 1 mL/min, did not significantly affect the measurement’s resolution characteristics. This confirms the excellent mass transfer properties of the core shell particle-based stationary phase. Therefore, the pH 7 buffer system was chosen and the flow rate was set to 1 mL/min for the following studies. Due to the fact that the resolution was already sufficient, no attention was paid to optimize the running conditions any further.

Under these conditions, several samples and the reference standard in different matrices were analyzed as shown in Fig. 1. The most abundant variant was allocated to the main variant and marked as K0, where K0 represents the complete cleavage of the C-terminal lysine. In the basic region, two peaks were evident, which caused incomplete lysine clipping, enumerated as K1 and K2, where one or both lysine residues remained attached to the heavy chain C-termini [21]. In the acidic area, there was only one pronounced peak (A1) evident for the standard. The anti-TNF-alpha antibody produced in our process revealed a different chromatographic pattern. The main variant (K0) was identified at a similar retention time, while the basic and the acidic region exhibited more variants. These differences are not remarkable due to the fact that the reference standard was a purified API, while the anti-TNF-alpha antibody expressed in a certain cell line with a defined media was produced under variable process conditions and not purified at all.

During repeated test sequences, it became apparent that slight variabilities in the buffer system resulted in minor retention time shifts caused by these sensitive gradients (see Fig. 1a, d). Consequently, the reference material was analyzed each time when a new buffer was prepared, both to control the performance characteristic and to ensure correct integration. To make the set of data comparable, the retention time was normalized. This was done by dividing the retention time *t*_*i*_ by the retention time of the main variant *t*_main_ peak. Thereafter, peak area integration was performed within a *t*_normalized_ of 0.76–1.23 for all analyses; in the following, this is referred to as the total peak area.

After the performance optimization, the reference standard and various samples were evaluated according different criteria. For the indication, whether or not the samples were affected by matrix proteins, both supernatant samples (*n* = 36) and standards (*n* = 46) were analyzed. The relationship between the total area and the amount of IgG, previously determined with BLI was assessed. The linear relationship of both indicated that the established performance was sufficient to obtain reliable data for standards and supernatants (see Fig. 1b).

Furthermore, for a comprehensive quantitative evaluation, it is important that the interprecision of the distribution is appropriate. The calculated coefficient of variation (CV) for the acidic, main and basic variants of the control standard (*n* = 15) was below 10% for all variants (see Table 2). These results are in accordance with the comprehensively verified data published by Lingg et al. [25]. For demonstrating the charge distribution reproducibility of the recombinantly produced mAbs in supernatants, as fed-batch samples, which were collected at day 4, were analyzed. Day 4 was at the end of the batch phase assuming that the experiments were performed in a similar manner. All temperature shifts were performed after the batch phase. The acidic and main variant distribution exhibited a CV of 4% and 10%, respectively (*n* = 8). The CV of the basic variant area was higher at 26%, caused by the low amount of this variant (see Table 2). Compared to the standard, the quantitative distribution of the fed-batch sample at day 4 was different. Acidic species were the most abundant variants, evidencing a possible impact of the chosen bioprocess conditions.

**Table 2:** Charge heterogeneity distribution consistency of the control standards and supernatant samples determined by the described CEX method using the pH 7 buffer system

Sample	Variant	Relative area ± *σ* (%)
Pharmaceutical standard (*n* = 15)	Acidic	16.2 ± 1.3
	Main	59.2 ± 1.6
	Basic	24.5 ± 0.8
Fed-batch samples drawn at day 4 (*n* = 8)	Acidic	69.2 ± 3.0
	Main	24.6 ± 2.5
	Basic	6.6 ± 1.7

Although it can be shown that a linear relationship between the total area and the amount of IgG exists, the variable amount of HCPs could falsify the chromatographic results, for instance, in the later stages of the cell culture process, when the viability decreases and the cells start to lyse. The HCP population is highly heterogeneously composed, but the majority should exhibit a pI below 7 and a molecular weight lower than 150 kDa [39, 40]. The ratio of HCP to IgG titer ranged between 20 and 50% and was independent of the feeding strategy (see Fig. 1c). Co-elution of any proteins or antibody fragments was not observed. Silver-stained fractionated samples revealed only one pronounced band at around 150 kDa (data not shown). Additionally, in the mock control, where the HCP content was even higher (up to 700 µg/mL), no additional peak occurred within the elution period of the mAb. In this respect, only a slight baseline drift of around 0.2–0.4 milli-absorbance units was detectable, primarily indicating an effect of supernatant compositions, which is not caused by HCPs. Spiking experiments in the mock control supernatant confirmed the assumption that HCPs do not affect the elution pattern (see Fig. 1d). Therefore, the contribution of HCPs and the culture supernatant matrix to the measured charge variant distribution was considered as not significant. Co-elution of any other proteins or other cell culture components did not affect the quality of the obtained data.

In summary, the results indicate that the determination of mAb charge distribution directly from supernatants is practicable. It has also been shown that the method is reliable and reproducible.

### Process monitoring of cell culture processes

The optimized method was used to monitor mAb charge heterogeneity during a cell culture process to elucidate the influences of temperature and glucose concentration.

**Fig. 2:**
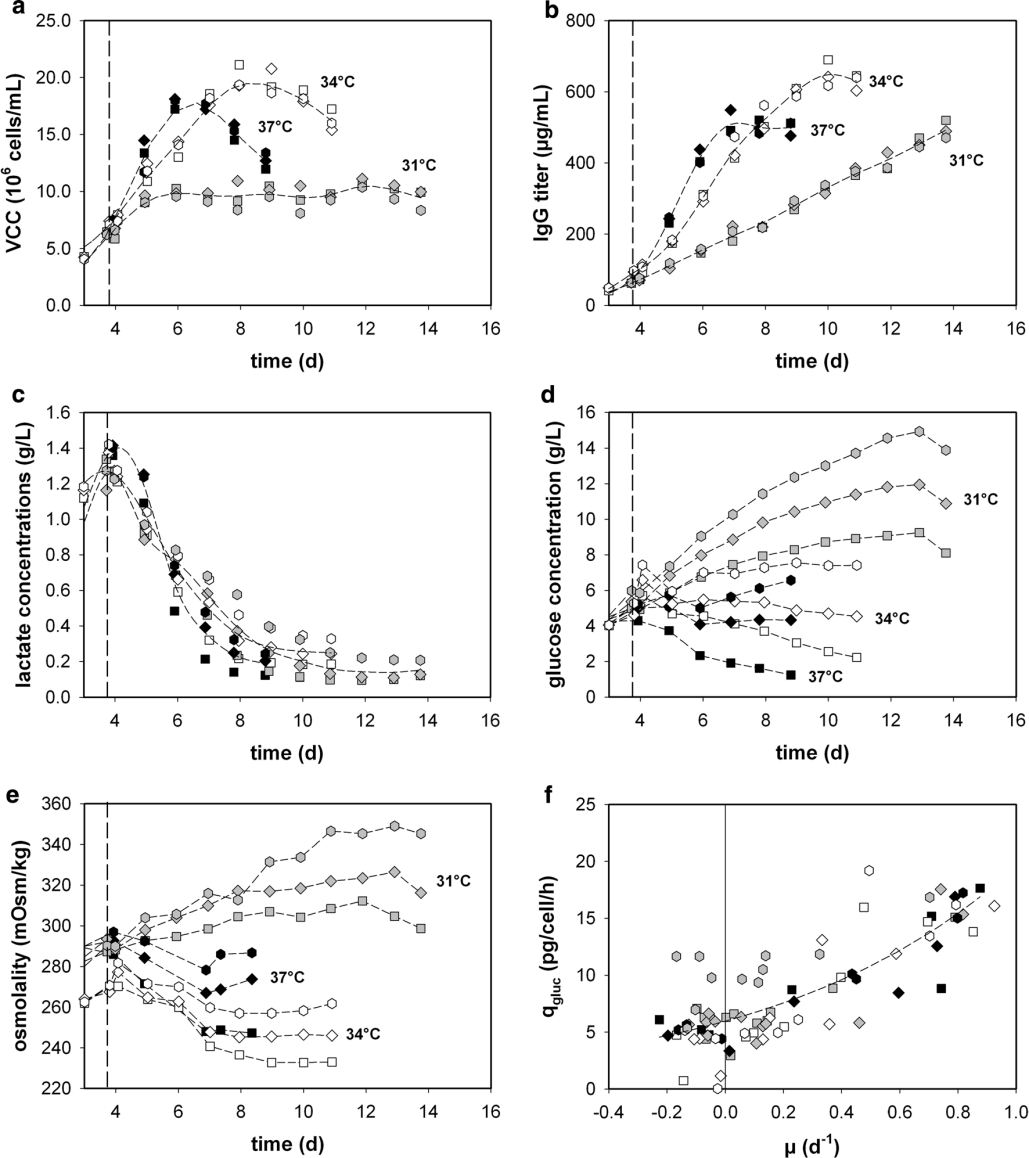
Fed-batch process parameters: **a** VCC, **b** protein titer, **c** lactate and **d** glucose concentration and **e** osmolality as a function of process time. **f** Specific glucose consumption (*q*_gluc_) as a function of the growth rate (*µ*). 37 °C + Feed 1 [filled square], 37 °C + Feed 2 [filled diamond], 37 °C + Feed 3 [filled hexagon], 34 °C + Feed 1 [unfilled square], 34 °C + Feed 2 [unfilled diamond], 34 °C + Feed 3 [unfilled hexagon], 31 °C + Feed 1 [grey square], 31 °C + Feed 2 [grey diamond], 31 °C + Feed 3 [grey hexagon]. Fed-batch process started at day 3. Vertical dashed line indicates temperature shift (≈ day 4). Short dashed lines indicate trends

The batch phase exhibited similar trends in all measured variables for each experiment. The process temperatures had a significant impact on overall productivity, growth rates and viability of the cells (see Fig. 2). Such observations have already been described in several publications [41–44]. Reduced process temperature can, moreover, be used for the proliferation control of cell culture processes [45]. Even though glucose concentrations in the cultures varied from 2 to 15 g/L (Fig. 2d), depending on the feed and temperature, almost no impact on the monitored process variables could be detected. Only the osmolality could be partly linked to the glucose concentration in the supernatant, which ranged from 345 (high glucose) to 233 mOsm/kg (low glucose) (see Fig. 2d, e). Lactate production was only observed in the batch phase, while consumption took place during the remaining process (Fig. 2c), independent of the temperature and glucose profile. Interestingly, no difference in specific nutrient uptake and byproduct formation rates was determined, due neither to the elevated glucose level nor to the change in process temperature. *q*_gluc_ was independent in respect of the feed or process temperature used (see Fig. 2f), with such behavior previously reported in other publications [46, 47]. The observation may be correlated with the fact that glucose uptake is not only dependent on the amount of glucose but also on amino acids such as leucine, lysine and serine [48].

**Fig. 3:**
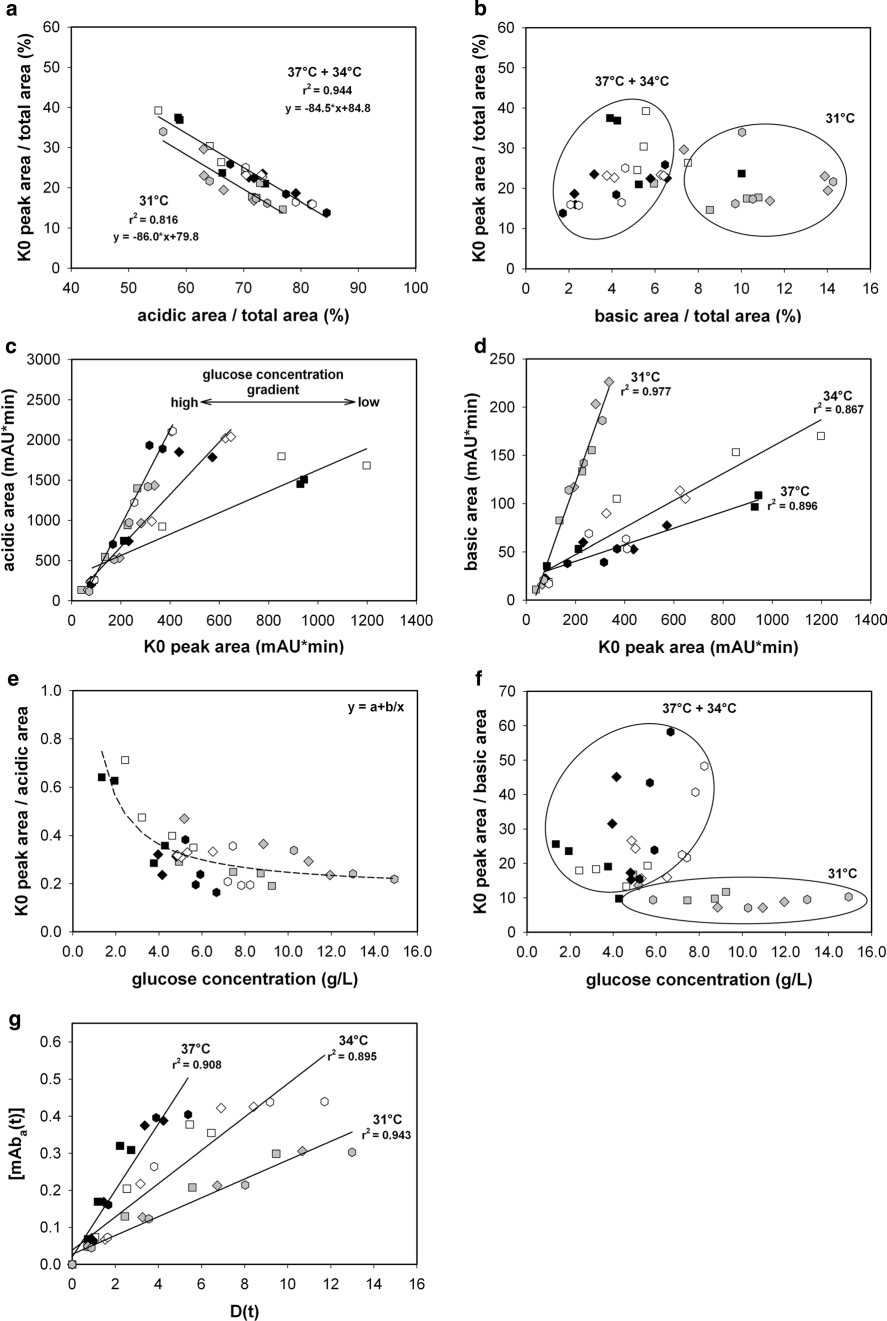
K0 main peak ratio as a function of the acidic (**a**) and basic variant (**b**) ratio. **c** Acidic area and **d** basic area as a function of the K0 main peak area. The ratio of the K0 peak area to the **e** acidic area and the **f** basic area as a function of glucose concentration. **g** Acidic peak area concentration at time point *t* as a function of *D*(*t*), that is, the product of newly built IgG concentration and glucose concentration integrated over time. 37 °C + Feed 1 [filled square], 37 °C + Feed 2 [filled diamond], 37 °C + Feed 3 [filled hexagon], 34 °C + Feed 1 [unfilled square], 34 °C + Feed 2 [unfilled diamond], 34 °C + Feed 3 [unfilled hexagon], 31 °C + Feed 1 [grey square], 31 °C + Feed 2 [grey diamond], 31 °C + Feed 3 [grey hexagon]

To evaluate the impact of process variation on charge distribution, several fed-batch samples from day 4, followed by samples after the temperature shift until the harvest criteria with a viability of 70%, were applied to the CEX column (*n* = 36). In turn, it became obvious that variation in the process parameters, glucose concentration and temperature affected the charge variant distributions to a great extent (see Fig. 3). Since the glucose concentration had no apparent influence on cell metabolism, it was supposed to have affected the mAb charge distribution in an extracellular manner (see Fig. 3a, c, e, g). This is also evidenced by the fact that the K0 main proportion correlates linearly with the percentage of acidic species (Fig. 3a). The resulting basic species are in opposition to this observation due to the fact that they mostly derive from incomplete C-terminal lysine processing, which is a known intracellular process (see Fig. 3b, d, f).

Under these defined process conditions, the basic species were generally rare, which suggests that C-terminal lysine processing occurred almost completely. However, process temperature predominantly affected the basic variant formation. As the amount of basic species was independent of the amount of the main variant, no trend could be identified; only two cluster regions were obvious (Fig. 3b). However, three linear relationships between the total basic area and K0 peak area could be determined by separating the data into three distinct groups according to the applied temperature (Fig. 3d). Evidently, lowering the temperature resulted in imperfect C-terminal lysine processing. This temperature-based occurrence was in accordance with previously published data [49]. The expression levels and the specific activity of the enzyme carboxypeptidase (B and H), which is considered to play a major role in C-terminal lysine cleavage, is temperature dependent [50, 51]. Hence, the processing of C-terminal lysine clipping, an important quality attribute [16], can be influenced by process temperature and sufficiently monitored with this method. The extracellular glucose concentration had no impact at all on basic species variations (Fig. 3f). The dataset could only be divided into two distinct groups: a 37 °C + 34 °C and a 31 °C cluster. No correlation was evident. Thus, in conclusion, the accumulated amount of basic species was the result of an intracellular process and most probably regulated by the amount and activity of the carboxypeptidase.

The vast impact of glucose on the micro-heterogeneity of the mAb was evident. The highest main variant (K0) content was observed at 34 °C with Feed 1 (low glucose). At an elevated glucose concentration, the amount of K0 was significantly reduced. For instance, the process at 31 °C with Feed 3 (high glucose) resulted in the highest charge heterogeneity. Acidic variants were the most abundant variants and ranged from 60 to 90% of the total peak area. It was obvious that an increase in the acidic species was attended by a decrease in the main variant (see Fig. 3a). The process at 31 °C, however, exhibited a slight parallel shifted linear correlation, due to the increased amount of basic species. At 31 °C, the proportion of basic variants was, on average, 5% higher than in the case of the other processes, which resulted in a decreased offset value of around the same proportion. However, both correlations exhibited a similar slope (Fig. 3a). The acidic heterogeneity was mainly dependent on the feed used; thus, an increase in glucose in the supernatant resulted in an enriched fraction of acidic variants (see Fig. 3c, e). It was lowest at 34 °C and 37 °C when a low glucose feed was applied. An induction of mAb alteration effects, due to osmolality and differences in the elution pattern, as reported by Schmelzer and Miller [52], was not evident. Acidic variant formation was mainly provoked by two parameters: the increasing total amount of main variants and incremental glucose concentration in the supernatant. Interestingly, regarding the latter, a saturation plateau was reached. At glucose concentrations higher than 7 g/L, the ratio of the total acidic area to the K0 main peak area remained almost unaffected (Fig. 3e).

According to these results, three assumptions were made: first, during those bioprocesses, acidic variants mainly evolved from the main ones already present in the supernatant; second, considering that the different feeds had no influence on any major process parameter, among others, biomass and productivity, the glucose concentration in the supernatant directly influenced the formation of acidic variants; and, third, there was a predefined number of possible glucose-inducible acidic variants. Under the selected process conditions, glycation, the non-enzymatic attachment of a reactive glucose to a protein, was most likely a feasible cause of acidic peak formation. It has already been related to the protein content and the glucose concentration [12] and also seems to be substantiated by this study.

Due to that fact that acidic species, induced by glucose, were most likely extracellularly formed, a second-order reaction, as proposed for typical non-reversible glycation reaction [53], was constructed. Unlike Yuk et al. [53], we set up some constraints. First, only a certain amount of the antibody is susceptible to be transformed into an acidic species. Second, if one antibody is not transformed within the certain time period, then a transformation will not occur during the rest of the process. We defined the second-order reactions as follow (Eqs. 1–3):

1 $$\left[ {\Delta {\text{mAb}}} \right] + \left[ {\text{glucose}} \right] \to \left[ {{\text{mAb}}_{\text{acidic}} } \right]$$

Setting up the second-order reaction in its differential form, as well as integrating and rearranging, yielded:

2 $$\left[ {{\text{mAb}}_{\text{acidic}} } \right]\left( t \right) = k \times D\left( t \right),$$

with

3 $$D\left( t \right) = \mathop \smallint \limits_{{t_{0} }}^{{t_{i} }} \left[ {\Delta {\text{mAb}}} \right] \times \left[ {\text{glucose}} \right] \times {\text{d}}t,$$

where [mAb_acidic_](*t*) represents the concentration of the acidic variants at time point *t*_*i*_, *k* is the reaction constant and *D*(*t*) is the integrated product at time point *t*_*i*_ of the newly built IgG and the glucose concentration. If the second-order reaction assumption is true, the plotting of [mAb_acidic_](*t*) against *D*(*t*) should yield in a straight line with a slope of *k* (Fig. 3g).

Evidently, the data are separated into three distinct groups, in respect of their applied process temperature. A linear regression was carried out. In conclusion, the reaction rate, and consequently the slopes of the representative lines, were dependent on the process temperature: 0.09, 0.04 and 0.03 $\frac{{g_{\text{acidic}} L}}{{g_{\text{IgG}} g_{\text{glucose}} d}}$, for 37 °C, 34 °C and 31 °C, respectively. These findings are in agreement with published research data. Typical antibody alteration effects, which result in acidic variant formation, for instance, deamidation [54, 55] or glycation [56, 57], can be modulated by temperature [51].

**Fig. 4:**
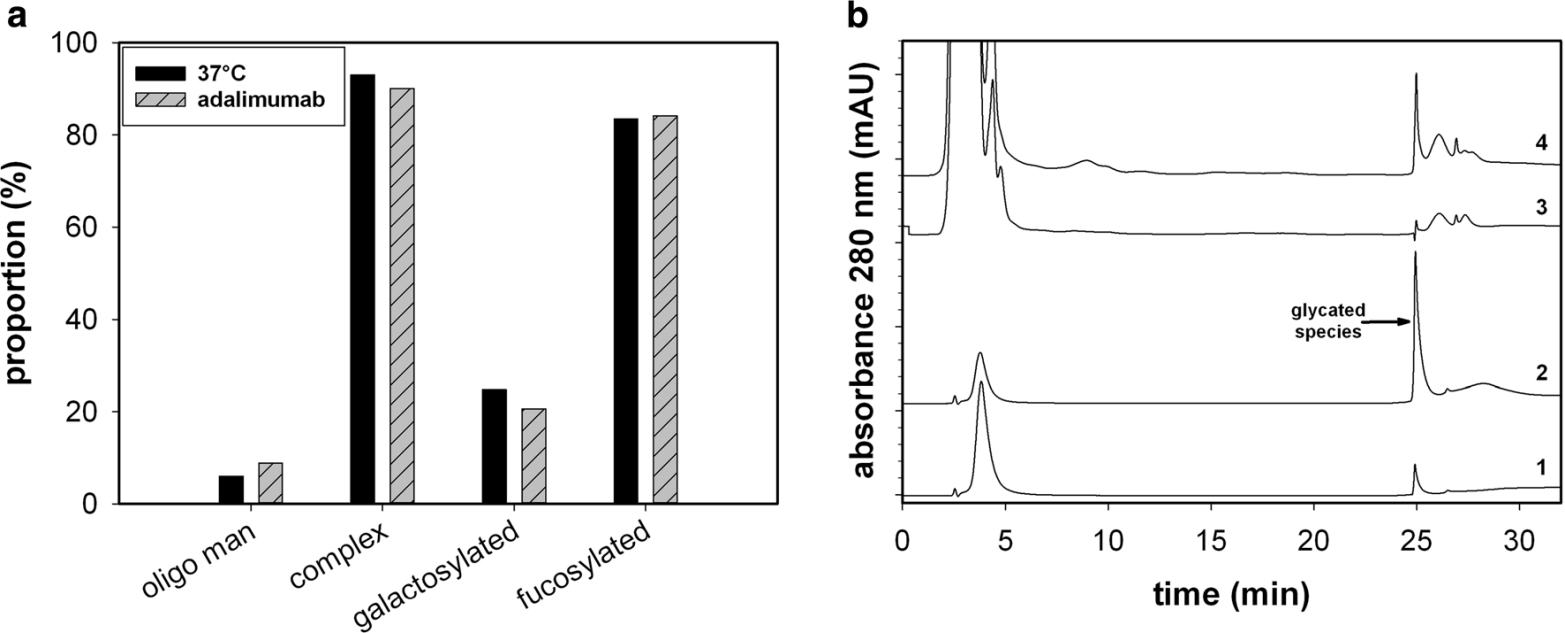
**a** Type of detected glycoforms for reference, for the recombinant produced mAb at 37 °C. **b** BAC chromatogram of the reference material (1), the reference incubated in 0.19 M glucose solution for 68 h at 37 °C (2), mocking supernatant from the host cell line (3), and a supernatant sample of a fed-batch process with a high number of acidic variants (≈ 90%) (4)

Finally, it can be speculated that the glycosylation pattern may also contribute to the acidic charge heterogeneity (Fig. 4a). Nevertheless, this is not the case, because anti-TNF-alpha antibodies do not contain any sialic acids, while the proportion of high mannose types is similar to the reference material [13, 21]. Additionally, the BAC analysis indicated that a substantial proportion of the mAb was glycated (Fig. 4b). The reference material, when incubated in a high glucose solution, exhibited a peak at a similar retention time. Thus, in conclusion, it was determined that glycation was the main driver for the formation of variable amounts of acidic species under the adjusted process conditions.

To the best of our knowledge, this is the first study that provides insights into the formation of charge variants during a cell culture process without using any pre-purification steps. Evidently, within the chosen process conditions, the formation of variable acidic species is of dominant importance. To achieve an understanding of consistent product quality, appreciating the mechanism of charge variant formation is inevitable. Rapid determination of the charge distribution pattern can significantly facilitate process optimization. This can be useful for process development and control of antibody and biosimilar production. Medium composition effects, such as glucose concentration and physical variations (e.g., temperature), on the generation of charge variants can be analyzed accordingly; thus, product quality attributes can be determined very early on in the process chain. That said, when assigning the occurring variants to distinct post-translational modifications, a more detailed and thorough characterization is necessary. The chromatographic pattern alone does not imply the occurred modifications [58]. Combination with other methods, such as LC–MS or BAC, could help to significantly improve the understanding of the mechanism of peak formation.

## Conclusion

In this work, we established a process-monitoring tool for the determination of charge heterogeneity of mAbs directly from cell culture supernatants. This method is based on cation exchange chromatography using a linear, basic pH gradient, with which cell culture supernatant samples can be directly analyzed for mAb charge heterogeneity without the need for prior protein purification. This represents a potential powerful tool for process development, since changes in product quality due to changes in process parameters can be detected earlier. It also has potential to serve as an in-process control method for any mAb production process, which can offer invaluable process knowledge. Under the Quality by Design paradigm, this increase in knowledge about the relationship of product and quality parameters can lead to a more consistent product quality through improved process control.

We also present a case study, in which the process parameter glucose level and temperature where analyzed for their effect on product quality. We showed that target protein production was predominantly affected by the process temperature, whereas the acidic variant formation and thus the product quality were highly influenced by the glucose level in the cell environment. The formation of acidic variants could be conclusively linked to an extracellular mechanism. These observations confirm the importance of the control of glucose level to ensure consistent high-quality mAb output. The process temperature, however, remains important as well, since, next to the titer, it secondarily affected the rate of acidic peak formation as well as basic variant formation.

However, charge heterogeneity of a mAb can be an adequate fingerprinting technique to confirm the desired product quality attributes, already early in the process chain. Depending on the mAb, the method can be adapted to gain information in an even shorter period of time. In general, this method will add great value to process optimization.
